# Liver Biochemistry During the Course of Influenza A/H1N1 Infection

**DOI:** 10.4021/gr551w

**Published:** 2013-07-14

**Authors:** Charalampos Seretis, Emmanuel Lagoudianakis, Nikolaos Salemis, Apostolos Pappas, George Gemenetzis, Fotios Seretis, Stavros Gourgiotis

**Affiliations:** a Department of Emergency Medicine, 401 General Army Hospital of Athens, Athens, Greece; b Second Department of General Surgery, 401 General Army Hospital of Athens, Athens, Greece; c Department of Internal Medicine, Argos Hospital, Argos, Greece; d Intensive Care Unit, 401 General Army Hospital of Athens, Athens, Greece

**Keywords:** Liver, H1N1, Influenza

## Abstract

Despite the multi-systemic effects of influenza A/H1N1 virus, the occurrence of hepatic injury during the natural course of the infection remains a matter of debate. We performed a review of the published clinical studies which assess the above mentioned relationship, reviewing the studies published in PubMed database (English literature), using the key words “H1N1”, “influenza A” and “liver”. We excluded case reports and clinical studies that referred to pediatric and transplanted patients, pregnants and patients with known history of chronic liver diseases. From a total of 96 results, a total of 78 papers met one or more of the exclusion criteria set. Evaluating the remaining 18 published papers, 14 more were excluded as they did not provide any sufficient data, relevant to the subject of our review. Although the analysis of the remaining studies revealed the existence of conflicting results concerning the exact degree and the potential mechanisms of liver injury in H1N1 positive patients, it can be assumed that influenza A/H1N1 virus is -or at least could be- a hepatotropic virus.

## Introduction

The outbreak of the influenza A/H1N1 virus in 2009 has undoubtedly been a major challenge for the health systems worldwide. As a result of its rapid spread and clinical severity, it was officially recognized by the WHO on the 11th of June 2009 as the first pandemic of the 21st century [1]. Since then, a huge amount of clinical studies have been published in order to elucidate the clinical manifestations that occur during the natural course of influenza A/H1N1 infection, providing sufficient data which highlight the multi-systemic effects of the virus [2]. Therefore, apart from the apparent impairment of the respiratory function and the immune system, influenza A/H1N1 has also proven to have a negative impact concerning the function of other vital organs, such as the liver, kidneys, myocardium, gastrointestinal tract and central nervous system [3-6]. When it comes to liver injury, autopsy findings in the liver in cases of fatal influenza A/H1N1 infection report the existence of extensive centrizonal haemorraghic necrosis of the hepatocytes, along with sinusoidal dilatation [7].

Focusing on the effect of this viral infection with respect to the liver function, there are relatively limited studies in the international literature that attempt to evaluate the latter, with the research topics spanning from retrospective reports of the alterations in liver biochemistry during the course of influenza A/H1N1 infection to pathology studies of the liver in patients who succumbed due to multiple organ dysfunction system, triggered by the virus. Without a doubt, in terms of laboratory essays, the values of the alanine aminotransferase (ALT), aspartate aminotransferase (AST), alkaline phosphatase (ALP), and gamma-glutamyltransferase (GGT) stand as the predominant screening examinations of the functional status of the liver [8]. Consequently, it would be justified to anticipate that the majority of the studies investigating the potential occurrence of liver injury as a result of the influenza A/H1N1 infection would primarily assess the above mentioned laboratory parameters. Herein, we will attempt to perform for the first time a comprehensive review of the published studies which examined the relationship between influenza A/H1N1 infection and liver dysfunction.

## Materials and Methods

We reviewed the studies published in PubMed database (English literature), using the key words “H1N1”, “influenza A” and “liver”. We excluded case reports and clinical studies that referred to pediatric and transplanted patients, as well as pregnants and patients with known history of chronic liver diseases (hepatitis, cirrhosis). The literature search was performed by three of the co-authors, working on an independent basis.

## Results and Discussion

We identified 4 (four) retrospective clinical studies, in which were documented the admission values of the commonly used liver enzymes.

One of them, performed by Chen Yingying [9], evaluated the frequency of abnormal liver chemistry in a cohort of 224 patients, who were tested positive for influenza A/H1N1 virus. The author mentions that all the patients were discharged within one week after the admission date, with undetectable H1N1 RNA after the treatment they received. Therefore, we could assume that on this occasion the percentage of the patients who experienced a severe infection would be rather low, fact that adds to the importance of the presented data, as liver involvement would have occurred virtually in the absence of the life threatening Multiple Organ Dysfunction Syndrome (MODS). In this study, an overall of 58% of the enrolled patients had abnormal liver tests, with pre-albumin being the most frequently affected laboratory parameter and was considered to be suggestive of a decreased capability of the liver in terms of protein synthesis. Concerning the routine liver chemistry indices, abnormal ALT, AST, ALP and GGT were reported in 13%, 9%, 6% and 19% of the total number of patients respectively. Certainly, one of the most significant limitations of the study is the lack of any association of the liver function parameters with the clinical symptoms, characteristics indicative of severity, inflammation markers and respiratory function tests of the study sample. However, it provided an adequate general profile of the frequency of abnormalities of the most common liver function tests in an unselected cohort of patients during the 2009 pandemic, highlighting the hepatotropic effects of influenza A/H1N1 virus.

Regarding the remaining three retrospective studies which assess the existence of a degree of liver injury as a result of the infection by the Influenza A/H1N1 virus, all of them attempt to compare the different extent of liver dysfunction between the seasonal influenza and influenza A/H1N1 during the pandemic of 2009. One of these three, performed by Papic et al [10] aimed specifically to describe the above mentioned differences, while the authors of remaining two studies, Zarogoulidis et al [11] and Wang et al [12], reported the different occurrence of abnormal values of the liver enzymes in the framework of a more general analysis of their patients’ laboratory findings at the point of admission. Despite their differences, all three studies indicate that liver injury was a more or at least equally frequent finding in H1N1-positive patients, as was the extent of the liver malfunction. Another common characteristic was the transient character of the liver injury, which was consistent with the severity of the infection and resolved at the stage of remission.

Specifically, Papic et al [10], comparing the laboratory results between their two subgroups, they reported that liver enzymes were significantly elevated in the pandemic group (AST: 35.78% versus 18.60%, ALT: 26.31% versus 7.36%, GGT: 36. 84% versus 16.47%). Moreover, the authors suggested a firm association of the liver enzymes elevation with the severity of hypoxemia; however, they mentioned that apart from transient and non-specific hepatic lesions, they did not encounter any cases of typical ischemic hepatitis. Also, the elevation of liver enzymes was positively correlated with the elevation of C-reactive protein, underlining the multi-systemic inflammation process, which was triggered by the viral infection. Finally, in the pandemic group, ALP was strongly correlated with the duration of the hospitalization, while, in the seasonal group, the duration of the hospitalization was correlated with all AST, ALT, ALP, as well as bilirubin.

On the contrary, Zarogoulidis et al [11], who also divided their patients in H1NI positive and H1N1 negative patients, failed to demonstrate statistically significant differences concerning the liver function between their study subgroups. However, their subgroups did not also significantly differ in terms of hypoxemia, as they did in the study of Papic et al, providing a potential explanation of these conflicting results, based on the assumption that one of the main pathogenetic mechanisms of liver injury is the development of a transient ischemic hepatitis. What is more, in the study of Zarogoulidis et al, only 10% of the total patients (12.1% of H1N1 positive and 7.4% of H1N1 negative patients) had abnormal values of liver enzymes at admission, which is far lower than the relevant percentages mentioned in the studies of Yingying and Papic et al [10, 11].

The study by Wang et al [12] appears to further complicate this puzzling conquest. Based on a similar pattern (H1N1 positive vs H1N1 negative), although the authors observed that hypoxemia was more severe in H1N1 positive patients, the latter was not accompanied by any similar differences with respect to values of the liver enzymes, battling the hypothesis of a transient ischemic hepatitis due to severe hypoxemia. Unfortunately, in this last study, the issue of liver involvement was scarcely evaluated by the authors and therefore very limited information, which would be relevant with the theme of our review, can be extracted.

### Conclusions

Considering all the results of the above mentioned studies, it becomes evident that one of the key reasons the influenza A/H1N1 pandemic tested the healthcare systems worldwide was the capability of the virus to impair the function of all vital organs and trigger the cascade of systematic inflammation, even in young patients [13]. What is more, it appears that the influenza A/H1N1 virus should be regarded as potentially hepatotropic, although the exact extent of the occurring liver injury cannot be at present safely evaluated. The attractive hypothesis of transient liver ischemia as a result of hypoxemia needs to be confirmed by further studies, which will implement more specific diagnostic tools to examine liver function. From our point of view, at least for the time being, probably the safest explanation of the mechanisms leading to liver dysfunction during influenza A/H1N1 infection stands exactly one step behind the establishment of hepatic ischemia and refers to the generalized stress due to the multi-systemic character of the infection, which is accompanied by a stormy release of cytokines that can cause direct liver injury per se [14, 15]. We strongly believe that the close interplay between liver function and the function of the immune system components will stimulate the researchers to re-evaluate the fluctuations of the parameters of liver function recorded in the national registries of the influenza A/H1N1-infected patients and provide a new insight in the multi-systemic effects of pandemic viral infections.
